# Machine Learning Framework for HbA1c Prediction: Data Enrichment, Cost Optimization, and Interpretability Through Stratified Regression and Multi-Stage Feature Selection

**DOI:** 10.3390/diagnostics16040607

**Published:** 2026-02-19

**Authors:** Mohamed Ezz, Majed Abdullah Alrowaily, Menwa Alshammeri, Alshaimaa A. Tantawy, Azzah Allahim, Ayman Mohamed Mostafa

**Affiliations:** 1 Computer Science Department, College of Computer and Information Sciences, Jouf University, Sakaka 72388, Saudi Arabia; malrowaily@ju.edu.sa (M.A.A.); mhalshammeri@ju.edu.sa (M.A.); 2 Information Systems Department, Faculty of Computers and Informatics, Zagazig University, Zagazig 44519, Egypt; eatantawi@fci.zu.edu.eg; 3 Information Systems Department, College of Computer and Information Sciences, Jouf University, Sakaka 72388, Saudi Arabia; akzallahim@ju.edu.sa

**Keywords:** HbA1c prediction, LightGBM, feature selection, stratified regression, cost-efficient biomarkers, interpretability, machine-learning, chronic-disease monitoring

## Abstract

**Background:** Measuring glycated hemoglobin (HbA1c) is essential for assessing long-term glycemic control, yet direct testing remains expensive and underutilized in many large-scale health surveys and resource-constrained settings. This study aims to (i) deliver a highly accurate and interpretable ML model for predicting HbA1c from routinely collected clinical, biochemical, and demographic data, (ii) reduce dependency on extensive laboratory panels by identifying a compact, cost-efficient subset of key predictors, and (iii) establish a transferable, explainable modeling framework applicable across chronic disease biomarkers. Unlike prior HbA1c prediction studies that focus primarily on classification or accuracy-driven models, this work introduces a unified framework for continuous HbA1c regression that jointly integrates cost-oriented feature parsimony, stratified regression validation, and explainability by design. **Methods:** We aggregated data from the National Health and Nutrition Examination Survey (NHANES) cycles 2007–2020, encompassing 66,148 records and 224 candidate features. We implemented a two-stage feature selection pipeline: Incremental Correlation Selection (ICS) to narrow the variable space, followed by Recursive Feature Elimination with Cross-Validation (RFECV) to isolate the most informative features. Model interpretability was assessed using partial dependence plots and feature importance analysis. **Results:** The optimal model, LightGBMRegressor with most-frequent imputation, achieved R^2^ = 0.7161, MAE = 0.334, MSE = 0.304, and MAPE = 5.56%, while using only 40 selected features. Interpretability analysis revealed clinically coherent relationships that align with physiological expectations. **Discussion:** The proposed framework maintains robust predictive performance while substantially reducing the number of required input features, enabling cost-efficient HbA1c estimation together with transparent, physiologically coherent model insights. By consolidating continuous HbA1c prediction, cost-aware feature selection, stratified evaluation, and explainability within a single pipeline are enhanced. **Conclusions:** This study advances beyond existing approaches and offers a practical blueprint for scalable biomarker estimation in population health and clinical decision-support applications. Its explainable, efficient, and generalizable design positions it as a strong candidate for clinical decision-support and population-health applications.

## 1. Introduction

Recent studies have demonstrated the feasibility of estimating HbA1c using machine-learning techniques applied to heterogeneous data sources, including lifestyle variables, electronic health records, longitudinal glucose measurements, and non-invasive physiological signals [1,2,3,4,5,6,7]. Collectively, this body of work confirms that surrogate-feature–based HbA1c modeling is clinically meaningful. Most existing approaches focus on classification or short-term prediction tasks rather than continuous HbA1c regression across population-scale cohorts; many rely on high-dimensional or costly feature sets that limit scalability, and interpretability is often treated as a post hoc analysis rather than being integrated into model design. These limitations highlight the need for modeling frameworks that explicitly balance predictive accuracy, feature parsimony, and explainability, particularly in large epidemiological datasets where HbA1c measurements may be incomplete.

In response to these challenges, we propose a machine-learning framework guided by three principal goals:

- Accurate and interpretable HbA1c prediction from routinely available clinical, biochemical, and demographic variables.
- Cost-optimized biomarker estimation, achieved by identifying a minimal yet high-performing subset of predictors, thereby reducing dependency on extensive laboratory panels.
- Explainable and transferable modeling architecture, enabling its application beyond HbA1c prediction into other chronic-disease biomarkers.

Although our framework additionally enables a fourth benefit—imputation of missing HbA1c values to enrich large-scale datasets—this study places strong emphasis on the modeling innovations: prediction accuracy, cost reduction, and interpretability. While point-of-care (PoC) devices provide rapid HbA1c measurements for individual clinical encounters, they represent single-time-point assessments that require physical testing. In contrast, the proposed framework focuses on modeling cumulative glycemic exposure as reflected by HbA1c using routinely available physiological and biochemical variables. This distinction enables retrospective estimation, large-scale epidemiological analysis, and dataset enrichment in scenarios where direct HbA1c measurement—whether laboratory-based or PoC—is unavailable or impractical. The data-enrichment function is discussed as a consequential, practical benefit rather than the primary innovation.

Our approach departs from prior work through the integrated design of feature selection, validation, and interpretability within a single regression-based framework. Firstly, we implement a two-stage feature-selection strategy: (i) Incremental Correlation Selection (ICS), which ranks features by their correlation with the target while reducing redundancy; followed by (ii) Recursive Feature Elimination with Cross-Validation (RFECV), which iteratively prunes features while optimizing performance. This layered approach offers computational efficiency and model interpretability. Secondly, we adopt stratified regression cross-validation for a continuous target, ensuring that each fold preserves the distribution of HbA1c across its range—thus improving generalizability and reducing bias in performance estimation. Thirdly, we benchmark multiple regression algorithms (including tree-based, linear-based, and boosting models) under consistent preprocessing and imputation scenarios, to identify the best trade-off between accuracy, parsimony, and interpretability. Finally, we embed explainability mechanisms into the workflow: feature-importance ranking, partial-dependence plots, and clinically meaningful variable ordering ensure the model is not a black box but a tool for insight as well as prediction. Accordingly, this work is structured around the following focused research questions:

**Q1:** To what extent can HbA1c be predicted accurately and interpretably from a reduced subset of routinely available clinical features?
**Q2:** What is the potential for cost-reduced biomarker estimation when the feature-set is minimized yet model performance remains robust?
**Q3:** How transferable and explainable is the proposed modeling pipeline for applications beyond HbA1c, in other chronic-disease biomarker contexts?

Machine learning (ML) and data-mining approaches have become foundational in diabetes research for disease detection, risk stratification, and biomarker modeling. However, when the target is continuous HbA1c estimation, as opposed to binary classification, far fewer studies deliver both high predictive accuracy and clinically useful interpretability in heterogeneous populations. Below, we review prior literature across five thematic streams: (a) diabetes classification and risk prediction, (b) HbA1c and glycemic biomarker estimation, (c) feature selection and interpretability in medical ML, (d) hybrid/ensemble and advanced modeling, and (e) explainable or longitudinal HbA1c modeling. We then summarize the gaps and position our contribution.

Diagnosing diabetes is crucial to lowering its rising prevalence. Various ML techniques, such as K-Nearest Neighbors, Support Vector Classifier, Logistic Regression, Gaussian Naive Bayes, and Random Forest, are used in medical science to analyze medical data and draw conclusions. The authors of [8] aimed to make use of the most important features, not all of them. They employed Logistic Regression after cleaning the data and selecting possible features. Their suggested method performed better than certain current methods that make use of ML models. The authors of [9] developed a computational approach that combines several forms of physical examination data to predict diabetes risk. A model based on eXtreme Gradient Boosting (XGBoost) was created to differentiate diabetic patients, yielding an AUC of 0.8768. Additionally, to enhance the model’s practicality and adaptability in clinical and real-world settings, a diabetes risk scorecard was developed using logistic regression to assess human health. The data were then statistically examined to determine the main variables affecting patients’ ability to manage their diseases. As presented in [10], an imbalanced dataset presents a challenge and needs to be balanced for diabetes prediction using multiple ML methods, such as Tomek and SMOTE. These outliers are also managed using the IQR method.

To evaluate and synthesize primary studies published in six digital libraries between 2000 and 2020, the authors of [11] carried out a systematic literature review. Thirty-two primary papers were chosen and examined in light of eight review questions. According to their findings, ensembles were more popular in recent years and generally outperformed single models. Nevertheless, a number of shortcomings in the ensembles’ building procedure and performance measures were found. They offered suggestions for creating accurate ensembles for predicting blood glucose levels. As presented in [12], a review of ten studies on diabetes prognosis employed diverse ML techniques, including CNN, SVM, RF, KNN, NB, ANN, GB, AdaBoost, and LR. Their findings demonstrated the growing reliance on ML in diabetes prediction, although performance varied across models. Building on this, the authors of [13] highlighted limitations in early diabetes identification, such as time consumption and poor feature selection accuracy. To address these, an improved Recursive Feature Elimination (RFE) method combined with a hybrid bagging classifier was proposed, leading to improved accuracy and specificity.

As proposed in [14], the discrepancy between HbA1c measurements and blood glucose-derived estimates was emphasized. Using clustering-based personalized models on real-world clinical datasets, K-means local nonlinear regressors (NLR) were applied to improve HbA1c estimation, achieving significantly lower mean absolute differences compared to general models. Similarly, the authors also explored boosting algorithms on the PIMA dataset, concluding that Gradient Boosting achieved the highest accuracy (92.85%) after comprehensive preprocessing and validation. An ensemble framework was later presented in [15], which introduced KFPredict by integrating a multi-input neural network with ML algorithms through soft voting. This method outperformed single classifiers, reaching 93.5% accuracy and substantially enhancing sensitivity and specificity. Likewise, a Clinical Decision Support System (CDSS) using the PIMA dataset and multiple classifiers has been developed [16]. By combining preprocessing, hyperparameter tuning, and a rule-based layer, accuracies above 90% for decision tree and histogram-based gradient boosting models were achieved and delivered through a web-based interface.

As presented in [17], an ensemble approach with AdaBoost for type II diabetes detection was applied, achieving 83% efficiency. A broader comparative analysis was carried out in [18], where a super-learner model reached 86% accuracy on the PIMA dataset and 97% on an early-stage diabetes dataset. Further advancements were made in [19], which introduced a tri-ensemble voting classifier with KNN imputation, attaining exceptional results—97.49% accuracy and 99.35% recall—demonstrating the importance of proper handling of missing data. Optimization-based approaches were presented in [20], which employed genetic algorithms for hyperparameter tuning and Particle Swarm Optimization (PSO) for data balancing. This framework showed substantial improvements in accuracy, AUC, and APR, with XGBoost yielding the most efficient results on CDC data. As presented in [21], the authors proposed an IoT-driven diabetes monitoring system integrating real-time data, hybrid optimization (K-means clustering and Sailfish Optimization), and deep learning (Bi-LSTM). Their model outperformed traditional methods, especially in imbalanced data settings. As presented in [22], a Deep Neural Network (DNN) framework was applied for diabetes risk assessment using biometric and clinical features, achieving an accuracy of 82%. Similarly, the authors also examined type II diabetes early detection using SVM, RF, and logistic regression on the PIMA dataset, where logistic regression achieved the best accuracy (79%) after hyperparameter tuning.

Explainable AI gained momentum in [23], where LIME and SHAP were leveraged for interpretability in logistic regression and random forest models trained on large-scale survey data. This approach reached 86% accuracy while highlighting model transparency. As proposed in [24], Natural Language Processing (NLP) was applied to EHRs of over 23,000 patients, predicting disease progression stages with up to 80% accuracy and identifying key determinants of metabolic deterioration. As presented in [25], the authors combined real-world data and RCT findings to predict HbA1c changes using various ML models, showing that follow-up models with dynamic patient data achieved superior results compared to baseline or RCT-only models. Large-scale studies were also conducted in [26], where ML models were trained on a dataset of over 556,000 medical examinations. XGBoost proved most effective, with 97.5% accuracy and 0.971 ROC-AUC. As presented in [27], the authors enhanced XGBoost through Bayesian optimization, achieving marginally better performance than grid search methods, reinforcing the algorithm’s potential for diabetes prediction and prevention. As presented in [28], the authors aimed to describe national trends in HbA1c among insulin-treated people with type 1 or type 2 diabetes (2009–2020 NHANES) and to quantify the proportions achieving ADA-recommended and individualized HbA1c targets by diabetes type and insulin regimen. As presented in [29], the authors aimed to quantify how often HbA1c-based and glucose-based measures give mismatched classifications of normoglycemia, prediabetes, and diabetes in the U.S. population (NHANES 2005–2016), and to assess the clinical implications of such misclassification. The authors of [30] aimed to evaluate how well the ADA/AAP pediatric clinical screening guideline identifies youth with prediabetes/diabetes based on biomarker-defined status in NHANES data, and to compare its performance with machine learning classifiers as a first step toward a simple, accurate questionnaire-based youth diabetes risk tool.

As proposed in [31], the authors aimed to compare multiple machine learning algorithms for predicting diabetes in adults using only lifestyle and easily obtainable variables (NHANES 2007–2018), in order to explore their potential as non-invasive tools for early diabetes risk detection. The literature shows substantive progress in classification and risk models, including NHANES-based ML studies, yet there remains a conspicuous gap for continuous HbA1c regression in large, representative cohorts. Prior HbA1c-oriented work often relies on specialized data (e.g., CGM users) rather than population-scale surveys, and interpretability is frequently applied post hoc without addressing outliers or heterogeneity. As proposed in [32], the authors developed a machine-learning framework for the detection and prediction of chronic diseases, focusing in particular on heart attack risk using the BRFSS dataset. They addressed the strong class imbalance through rigorous data cleaning, feature selection, and hyperparameter tuning of models such as LightGBM, XGBoost, and logistic regression, ultimately building an ensemble that achieved improved balanced accuracy and recall for high-risk cases, demonstrating the value of ML-based early risk stratification in cardiovascular disease.

As explained in [33], the authors proposed a novel approach that combines layer-weighted attention with an ascending feature selection strategy to predict the seriousness level of adverse drug events using FAERS data. By jointly modeling NLP-derived representations of active substances and structured demographic/event features, and progressively adding the most informative variables, their method achieved strong performance and highlighted how carefully designed attention mechanisms and feature selection can enhance pharmacovigilance risk prediction. As presented in [34], the authors provided an innovative framework that employs tailored semantic embedding together with recent ML models to predict the seriousness of drug–drug interactions based on FAERS reports. Using advanced biomedical word vectors and powerful classifiers such as CatBoost, their approach captures subtle relationships between co-administered drugs and associated outcomes, enabling more precise assessment of DDI severity for clinical decision support and pharmacovigilance.

We address these limitations by centering the task on continuous HbA1c prediction from NHANES 2007–2020, combining a two-stage feature selection pipeline—Incremental Correlation Selection followed by RFECV—with a stratified regression cross-validation design that balances performance across the HbA1c spectrum. To strengthen interpretability beyond standard post hoc tools, we introduce Enhanced Partial Dependence Plots (EPDPs), which restrict attention to informative value ranges and dampen outlier influence, yielding clearer and more clinically faithful marginal effects. In contrast to classification-oriented systems and physiology-aware frameworks that do not target continuous HbA1c on population data, our approach unifies robust selection, high-fidelity regression, and refined interpretability in a single, reproducible pipeline. As presented in Table 1, a list of abbreviations for the whole paper is included.

In summary, existing research provides strong foundations in diabetes classification, ensemble modeling, and explanation techniques, but lacks a cohesive, interpretable pipeline for continuous HbA1c prediction on NHANES scale cohorts. To facilitate transparent comparison, the next section (Section 2) provides a detailed description of the dataset used in this study (NHANES 2007–2020), including cohort characteristics, feature composition, preprocessing procedures, and feature statistics. Section 3 explains the full methodology—data preparation, two-stage feature selection, model training, and evaluation protocol. Section 5 presents experiments and results. Section 4 discusses the findings by explicitly answering the research questions. Section 5 concludes with contributions, limitations, and directions for future work.

## 2. Materials and Methods

This study uses data from the National Health and Nutrition Examination Survey (NHANES) cycles 2007–2020, a rich, nationally representative dataset of the U.S. civilian non-institutionalized population that integrates detailed clinical, laboratory, anthropometric, and questionnaire data. The central outcome of interest is HbA1c (variable LBXGH in NHANES)—a proven marker of long-term glycemic exposure. NHANES is a repeated cross-sectional survey conducted in biennial cycles rather than a continuous or longitudinal study. Each participant contributes a single examination record per survey cycle, with clinical assessments, laboratory measurements, and questionnaires collected at the time of enrollment.

### 2.1. Data Sources and Consolidation

We merged multiple NHANES components—including demographics, examination (physiological/anthropometric) data, laboratory biochemistry panels, complete blood-count and urine/kidney metrics, lifestyle questionnaires, and environmental exposures—across cycles. Harmonizing variable names, units, and definitions across cycles was essential. The initial combined dataset comprised 66,148 participant-records and 224 numeric candidate features spanning diverse health-related domains. At the raw dataset level, the merged NHANES cohort comprised 66,148 participant records. Participant ages spanned a wide range, with a median age of approximately 28 years (interquartile range: 10–54 years), reflecting the broad population coverage of NHANES. Sex distribution was approximately balanced (male: 33,355; female: 32,793).

### 2.2. Feature Categorization

To structure the analysis and support cost-efficiency considerations, features were organized into nine coherent categories: Demographics; Anthropometry (including BMI and waist circumference); Blood Pressure; Complete Blood Count (CBC); Biochemistry (such as glucose, lipids, albumin-creatinine ratio, etc.); Kidney/Urine Measures; Environmental/Exposure Markers; Lifestyle/Questionnaire Measures; and Derived Ratios (for example waist-to-hip or lipid ratio variables). This categorization enabled targeted missing-value inspections, facilitated analysis of cost-proxy features, and shaped the subsequent filtering and selection strategy. Collectively, these feature categories capture a broad spectrum of medical conditions and comorbidities relevant to metabolic and cardiovascular health, including markers related to glucose regulation, lipid metabolism, adiposity, renal function, blood pressure, inflammation, and lifestyle-associated risk factors. The dataset is predominantly composed of numerical variables, including laboratory measurements, anthropometric indices, and physiological indicators. The final feature set includes both continuous numerical variables and discrete categorical variables derived from NHANES demographic, socioeconomic, and survey-administration fields. Categorical variables include sex, race/ethnicity, education level, marital status, citizenship, household composition, income-related indicators, language and proxy-response variables, survey cycle identifiers, and selected clinical status indicators (e.g., blood-pressure status). In total, 33 categorical variables were included and encoded during preprocessing, while the remaining features consist of continuous laboratory measurements, anthropometric indicators, and physiological variables.

### 2.3. Data Cleaning and Missing-Value Filtering

Initial exploration revealed that roughly 48.6% of cells in the feature matrix were missing. No assumption of missing completely at random (MCAR) was made for the NHANES data. Given the survey-based nature of NHANES, missingness may depend on participant characteristics, examination protocols, or laboratory subsampling, and is therefore more plausibly missing at random (MAR) or missing not at random (MNAR). To mitigate potential bias arising from non-MCAR missingness, we applied conservative feature-level filtering, removed variables with excessive missingness, and performed imputation strictly within the cross-validation framework to avoid information leakage. To preserve analytic robustness and avoid instability from sparsely populated features, any variable with more than 60% missing entries was removed—a pragmatic threshold commonly used in large-scale observational and machine-learning studies to exclude features with excessive missingness, where the exact cutoff is chosen to balance information retention, model stability, and bias–variance trade-offs. to reduce bias and ensure model reliability. While no universal missing cutoff exists, prior methodological work consistently recommends removing features with very high proportions of missing values to avoid unstable imputation and unreliable model estimates in population-scale datasets [35,36]. After eliminating high-missing features, records lacking a measured HbA1c value were excluded solely for supervised model training and evaluation, as a ground-truth target is required for regression-based learning and unbiased performance assessment. This resulted in an analytic sample of 42,208 participants with 125 numeric features. Importantly, the exclusion of participants without measured HbA1c values was limited to the supervised training and validation phases. Once the model was robustly trained using observed HbA1c values, it can be applied to estimate HbA1c for records where laboratory measurements are missing. This separation between model development and downstream application avoids target leakage and preserves methodological validity, while still enabling dataset enrichment through post-training HbA1c estimation.

### 2.4. Imputation and Pre-Processing

Among the remaining features, four numeric imputation strategies were compared: no imputation (row exclusion), mean substitution, median substitution, and most-frequent substitution. All imputation and scaling (z-score standardization) were performed within the cross-validation framework to avoid leakage.

### 2.5. Final Feature-Set

**Figure A1:**
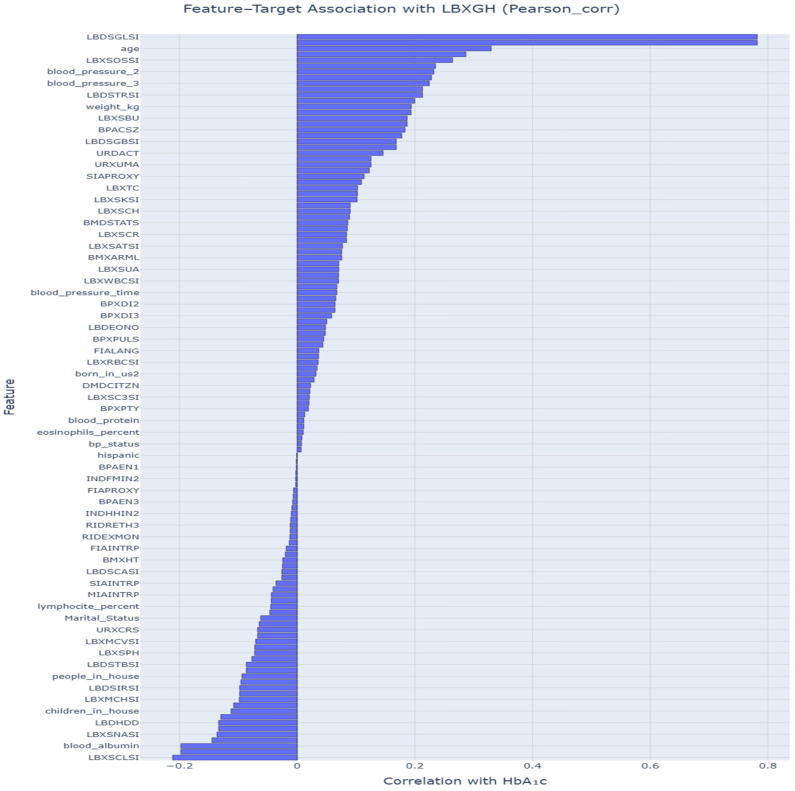
Correlation features for LBXGH levels.

**Figure A2:**
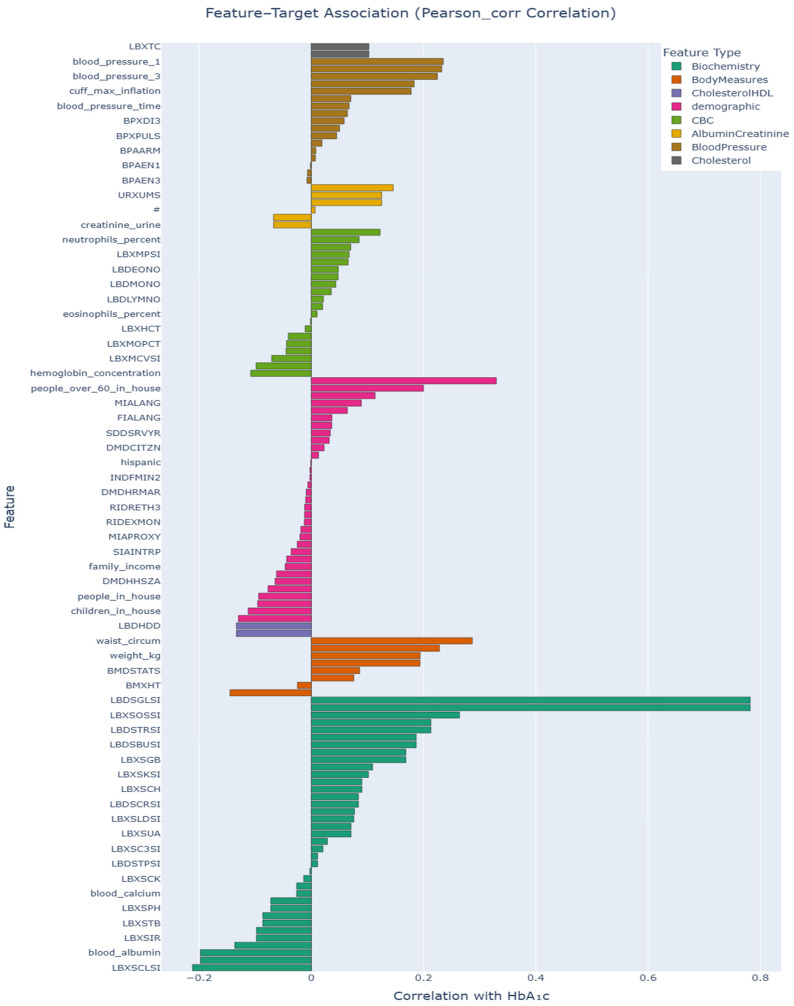
Feature Target Association.

From the cleaned set of 125 numeric features, our two-stage feature-selection pipeline produced a final predictive subset of 40 features. Pearson correlation was retained as the primary reported metric, as it directly reflects linear associations relevant to regression-based modeling, while Spearman and Kendall correlations were evaluated only as robustness checks to assess sensitivity to non-linearity and outliers. A comprehensive list of health-related variables and their characteristics is provided in Appendix A Table A1, and the extended correlation visualizations are provided in Appendix A Figure A1 and Figure A2.

Our proposed methodology provides an advanced machine-learning framework, explicitly designed to achieve three interconnected objectives:

- Accurate and interpretable HbA1c prediction using routinely collected clinical and biochemical features.
- Cost-efficient biomarker estimation by identifying a minimal but highly informative set of predictors.
- A transferable and explainable modeling architecture that can be applied beyond HbA1c to other chronic-disease biomarkers.

The innovation of this framework comes from the integration of preprocessing, dual-stage feature-selection, model benchmarking, stratified regression validation, and explainability tools into a cohesive pipeline. Each stage is carefully aligned to one or more of the objectives, rather than being treated as an afterthought.

### 2.6. Workflow Overview

**Figure 1:**
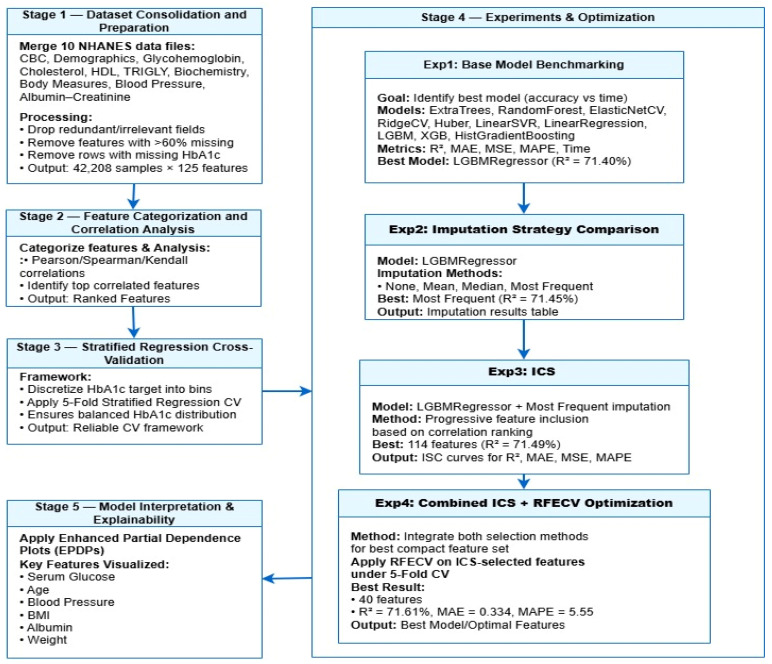
Workflow for proposed HbA1c prediction.

As illustrated in Figure 1, our workflow proceeds through the following major phases:

1. Data consolidation and cleaning
2. Preprocessing and imputation strategy comparison
3. Dual-stage feature-selection (combining filter and wrapper methods)
4. Model benchmarking via stratified regression cross-validation
5. Interpretability and explainability analysis

What elevates this methodology is that feature-selection is not isolated; it is co-designed with model performance and interpretability in mind. Likewise, the model benchmarking is informed by cost-efficient feature use, and the explainability layer is embedded rather than appended.

### 2.7. Model Benchmarking (Experiment 1)

We comparatively assess a suite of regression algorithms: ExtraTreesRegressor, RandomForestRegressor, RidgeCV, HuberRegressor, LinearSVR, LinearRegression, XGBoostRegressor, and LightGBMRegressor. Each algorithm is evaluated using stratified-5-fold regression cross-validation (see Section 3.5) to maintain representativeness of the HbA1c target distribution. The selected algorithm must not only deliver high accuracy but also operate within the compact-feature, interpretable framework we target. Hyperparameter optimization was not performed extensively for individual models. Instead, all regression algorithms were evaluated using standard or commonly adopted default configurations to ensure fair comparison and reproducibility across experiments. This design choice was intentional, as the primary focus of the study is on the impact of feature selection, cost-oriented parsimony, and evaluation strategy rather than aggressive model-specific tuning.

### 2.8. Imputation Strategy Optimization (Experiment 2)

Large-scale survey datasets such as NHANES automatically involve missing values. To maximize cost-efficiency and model robustness, we evaluate four numeric imputation strategies: mean substitution, median substitution, most-frequent substitution, and no substitution (using features as they appear). Each strategy is nested within the cross-validation folds to prevent leakage. The chosen strategy is the one that optimizes the metrics (R^2^, MAE, MAPE), supporting our cost-efficient biomarker estimation objective.

### 2.9. Dual-Stage Feature-Selection Strategy (Experiments 3 and 4)

Our key innovation lies in the two-stage feature-selection pipeline, combining filter and wrapper approaches to deliver a compact, high-signal predictor set that meets cost- and interpretability-goals. Feature selection was conducted within the cross-validation framework to avoid information leakage. Specifically, the two-stage feature-selection process (Incremental Correlation Selection followed by RFECV) was applied independently within each training fold, and the resulting feature subset was then used to evaluate performance on the corresponding held-out test fold. No feature information from the test data was used during feature selection. The final feature set reported in the manuscript corresponds to the configuration that consistently yielded the best aggregate out-of-fold performance and is presented for interpretability and deployment considerations.

#### 2.9.1. Stage 1—Incremental Correlation Selection (ICS)

ICS is a filter method that ranks each candidate feature $X_{i}$ according to its linear correlation with the target variable HbA1c (Y). Only those features with strong individual association and minimal redundancy progress to the next phase. This supports both cost-efficiency and interpretability as in Equation (1):

(1) $$r_{X_{i},Y}=\frac{\sum_{j=1}^{n}\;\left(x_{i\;j}-{\bar{x}}_{i}\right)\left(y_{j}-\bar{y}\right)}{\sqrt{\sum_{j=1}^{n}\;{\left(x_{i\;j}-{\bar{x}}_{i}\right)}^{2}}\sqrt{\sum_{j=1}^{n}\;{\left(y_{j}-\bar{y}\right)}^{2}}}$$

where ${\bar{x}}_{i}$ and $\bar{y}$ are the means of feature i and the target, respectively. We then rank features in descending order of $|r_{X_{i},Y}|$. The ICS Algorithm is based on the following steps:

- Compute correlation for each feature with HbA1c.
- Sort features by absolute correlation value, highest first.
- Incrementally build subsets by adding features one by one (from highest correlation downward), train a model for each subset, evaluate the performance (e.g., R^2^), and stop when performance no longer improves significantly as explained in Equation (2).

(2) $$R-squaredR^{2}=1-\frac{\sum_{j=1}^{n}\;{\left(y_{j}-{\hat{y}}_{j}\right)}^{2}}{\sum_{j=1}^{n}\;{\left(y_{j}-\bar{y}\right)}^{2}}$$

where ${\hat{y}}_{j}$ are the predicted values. The value $k_{ICS}^{*}$ that maximizes R^2^ determines the candidate subset size from ICS.

#### 2.9.2. Stage 2—Recursive Feature Elimination with Cross-Validation (RFECV)

The RFECV is a wrapper method that, starting with the subset produced by ICS, iteratively removes the least-important features (as determined by the model’s importance measures) and uses cross-validation at each elimination step to find the optimal subset. This supports model interpretability and cost-efficient estimation in Equation (3).

(3) $$k^{*}=\arg\underset{k\in \left\{m,\ldots ,n\right\}}{\max}s_{k},S^{*}=\left\{f_{1},\ldots ,f_{k^{*}}\right\}$$

where $s_{k}$ = cross-validated score (e.g., R^2^) for a model trained with k features, n is the starting number of features, and m is the minimum number of features allowed. The RFECV Algorithm is explained as follows:

- Begin with the $k_{ICS}$ features from Stage 1.
- For each k from $k_{ICS}$ down to m: train the model using stratified CV, compute score $s_{k}$.
- Remove the feature with the lowest importance.
- Continue until the optimal $k^{*}$ is found (the highest average CV score).
- Return $S^{*}$, the feature set of size $k^{*}$.

#### 2.9.3. Integrated Algorithm—Combined ICS + RFECV

This integrated algorithm (Algorithm 1) directly addresses cost-efficiency (minimal, high-signal features) and interpretability (transparent selection process, model-based elimination).
**Algorithm 1.** Combined AlgorithmInput full feature set F, target HbA1c Y, estimator M, CV folds.Compute $|r_{X_{i},Y}|$ for each feature $X_{i}\in F$.Rank features by descending $|r_{X_{i},Y}|$.Incrementally add features from the highest rank onward; for each subset size k, train M, compute R^2^; select $k_{ICS}$ that gives maximal R^2^.Set current feature subset S← top $k_{ICS}$ features.For *k* = ∣S∣ down to m:
○Train M using S with stratified CV; compute score $s_{k}$.○Identify and remove the feature in S with the lowest importance.Let $k^{*}$ = value of k with highest $s_{k}$; return $S^{*}$ = features at $k^{*}$.

### 2.10. Stratified Regression Cross-Validation

Traditional k-fold CV may lead to uneven representation of the continuous target variable across folds. Stratified regression CV mitigates this by discretizing the target variable (HbA1c) into quantile-based bins and ensuring each fold contains samples from each bin. This improves performance estimation reliability across the full range of the target, as explained in Equation (4).

(4) $${\bar{M}}_{\mathrm{strat}}=\frac{1}{K}\overset{K}{\underset{i=1}{\sum }}\;M\left(f_{\mathrm{train}\left(-i\right)}\left(X_{-i}\right),Y_{i}\right)$$

where K = number of folds, $f_{\mathrm{train}\left(-i\right)}$ is the model trained on all but fold i, and $Y_{i}$ are the true targets in fold i. The Stratified Regression Algorithm is explained as follows:

- Partition the continuous HbA1c values into Q quantile-bins.
- Create K folds such that each fold has proportional representation from each bin.
- For each fold: train the model on the other folds, validate on the current fold; compute metric $M_{i}$.
- Average the metrics across folds to get ${\bar{M}}_{\mathrm{strat}}$.

Only records with observed HbA1c values were included during model training and cross-validation, while HbA1c estimation for records without laboratory measurements is treated as a post-training application of the finalized model.

Stratified regression cross-validation was adopted to ensure that each fold preserves the distribution of HbA1c values across its range. Unlike standard k-fold cross-validation, which may produce imbalanced folds when the target variable is continuous and skewed, stratified regression reduces evaluation bias and improves stability by maintaining comparable target distributions in training and test sets. Group cross-validation was not applicable in this setting because NHANES data are cross-sectional, with no repeated measurements or subject-level grouping that would otherwise require group-wise partitioning to prevent information leakage.

### 2.11. Interpretability and Explainability

Aligned with our objective of a transferable, explainable modeling architecture, for the final selected model, we compute:

- Feature importance vector w or tree-based importance scores.
- Partial dependence plots (PDP): for feature $X_{i}$,
  (5) $$D_{i}\left(x\right)=E_{X_{-i}}\left[\hat{Y}|X_{i}=x\right]$$
- SHAP values: $\phi_{i}=\mathrm{Shapley}(X_{i},\hat{Y})$.

These tools convert predictive power into interpretive insight, enabling clinicians and researchers to understand how each variable drives HbA1c.

### 2.12. Evaluation Metrics and Validation

Model performance is assessed using: coefficient of determination (R^2^), mean absolute error (MAE), mean squared error (MSE) and mean absolute percentage error (MAPE). All results derive from stratified-5-fold cross-validation and are reported as fold-averaged values. Residual analyses and training-validation loss curves are inspected to detect potential overfitting or heteroscedasticity.

(6) $$\mathrm{MAE}=\frac{1}{n}\overset{n}{\underset{j=1}{\sum }}\;|\;y_{j}-{\hat{y}}_{j}\;|,\mathrm{MAPE}=\frac{1}{n}\overset{n}{\underset{j=1}{\sum }}\;\frac{|\;y_{j}-{\hat{y}}_{j}\;|}{y_{j}}$$

In summary, the methodology we present weaves together preprocessing, imputation, dual-stage feature selection, robust model benchmarking, and interpretability into a coherent, goal-driven pipeline. By integrating the filter-based ICS and wrapper-based RFECV into one unified algorithmic flow, applying stratified regression cross-validation, and embedding explainability tools from the start, our framework moves beyond traditional modeling frameworks to a purpose-built architecture for accurate, cost-efficient, and transparent HbA1c prediction. This methodological design not only underpins our current study but also lays the foundation for future extensions into other chronic-disease biomarkers, ensuring the research is both scientifically rigorous and practically deployable.

## 3. Results

This section presents the full results of our experiments, structured around four consecutive experiments (Exp 1–Exp 4) that progressively refined the predictive framework for HbA1c estimation. All experiments were performed using 5-fold Stratified Regression Cross-Validation, where the continuous HbA1c target was discretized into quantile bins to ensure each fold maintained the distributional balance of the target. This stratified design was critical to avoid overfitting and to guarantee that each train/test partition adequately represented the variability of the population. The evaluation metrics used were: coefficient of determination (R^2^), mean absolute error (MAE), mean squared error (MSE), and mean absolute percentage error (MAPE).

### 3.1. Experiment 1: Model Benchmarking

**Figure 2:**
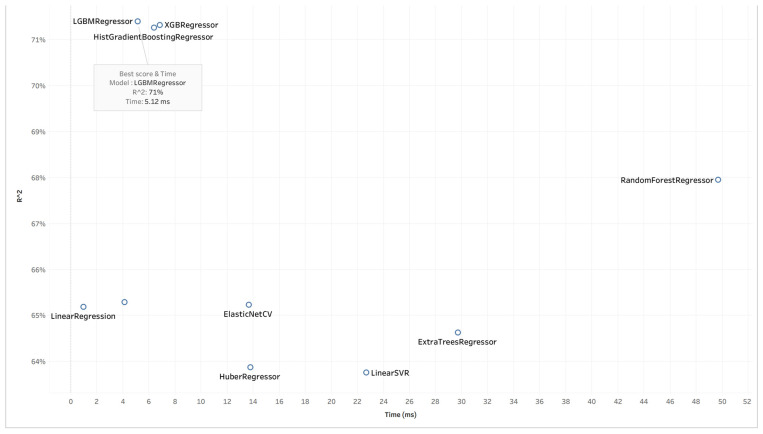
Base Models Comparison (R^2^ vs. Time).

The first experiment explored multiple regression algorithms to identify the model that achieves the best performance-time trade-off. The algorithms compared include ExtraTreesRegressor, RandomForestRegressor, ElasticNetCV, RidgeCV, HuberRegressor, LinearSVR, LinearRegression, LGBMRegressor, XGBRegressor, and HistGradientBoostingRegressor. Each model was trained with all available numeric features (*n* = 124) using 5-fold stratified regression CV. The results are summarized in Table 2 and illustrated in Figure 2, which plots model R^2^ versus computational time. Time represents the average end-to-end training time per cross-validation fold (including preprocessing, model fitting, and prediction).

The LGBMRegressor achieved the best overall performance (R^2^ = 71.40%), combining high predictive accuracy with efficient computation time (≈5.1 s). This model was selected for all subsequent experiments. This experiment focuses on cross-sectional predictive robustness rather than temporal forecasting and evaluates how different imputation choices affect out-of-sample regression performance.

### 3.2. Experiment 2: Imputation Strategy Evaluation

Based on the selected LGBMRegressor, we evaluated different imputation strategies applied exclusively within each training fold to prevent leakage, as explained in Table 3. The four strategies compared were: None, Mean, Median, and Most Frequent.

The *most frequent* imputation strategy slightly improved R^2^ (71.45%) and minimized MSE, indicating better handling of missing categorical-like numeric patterns. Although the absolute performance differences are small, this strategy provides the most stable aggregate out-of-fold performance and was thus selected for all following experiments.

### 3.3. Experiment 3: Incremental Correlation Selection (ICS)

In this experiment, Incremental Correlation Selection (ICS) was applied to progressively add features according to their absolute Pearson correlation with HbA1c. At each iteration, performance metrics were recalculated, and the process stopped when no further improvement was achieved.

**Figure 3:**
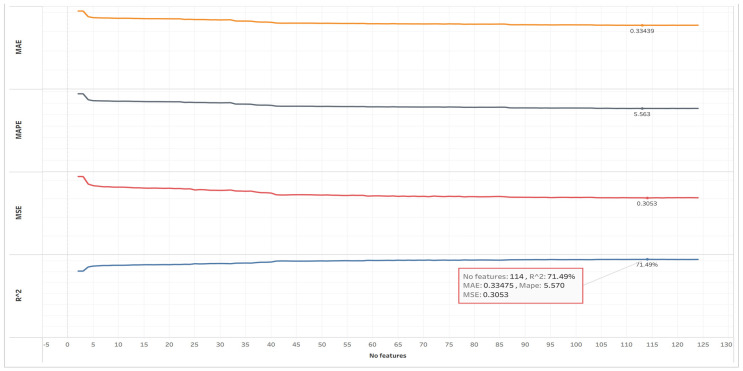
ICS performance curves (R^2^, MAE, MAPE, and MSE).

Figure 3 illustrates how predictive performance evolves as additional features are incorporated in a cross-sectional setting, highlighting diminishing returns beyond a certain feature count. The best performance was achieved with R^2^ = 71.49% at 114 features, as shown in Table 4.

The ICS experiment demonstrated that most predictive information can be captured by roughly 90% of the features, and that incremental addition beyond 114 variables adds negligible gain. This result motivates subsequent feature refinement aimed at reducing complexity without sacrificing predictive fidelity.

### 3.4. Experiment 4: Combined ICS + RFECV Feature Refinement

**Figure 4:**
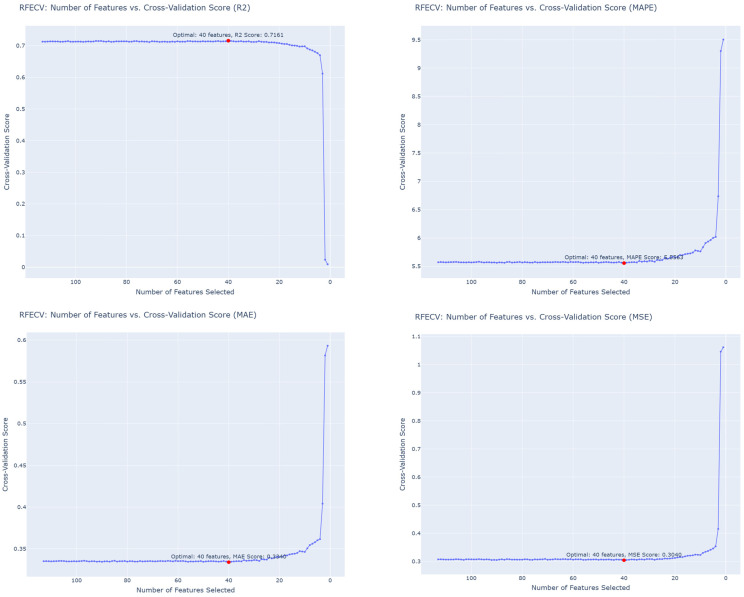
RFECV Results for R^2^, MAPE, MAE, and MSE.

The final experiment integrated ICS filtering with RFECV wrapper refinement to identify the minimal feature subset providing the best generalization. Figure 4 presents the performance trajectories as features were recursively eliminated. The optimal configuration was achieved with 40 features, yielding R^2^ = 71.61%, MAE = 0.334, and MAPE = 5.55% as explained in Table 5.

The combination of ICS and RFECV provided a compact, high-signal feature set with reduced complexity and maintained predictive performance compared with models using all available features. Although the absolute performance differences across the experimental settings are modest, Exp4 achieves comparable out-of-sample predictive accuracy while substantially reducing the number of required features. Accordingly, Exp4 is selected as the final configuration primarily due to its improved feature parsimony, cost efficiency, and deployment practicality, rather than a large performance margin.

### 3.5. Model Validation and Distribution Alignment

**Figure 5:**
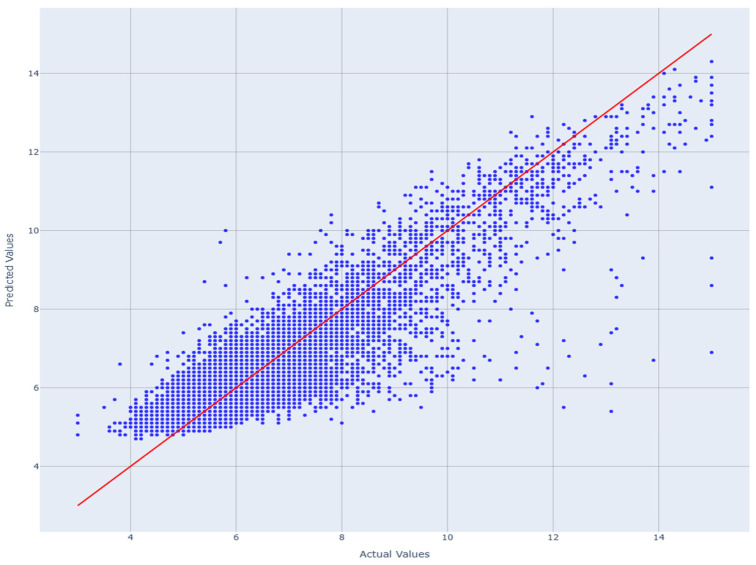
Scatter plot of aggregated out-of-fold predicted versus actual HbA1c values across five-fold stratified cross-validation. Each point represents a held-out test sample from one cross-validation fold. The red identity line indicates perfect agreement between predicted and observed values; deviations from this line reflect prediction error and bias across the HbA1c range.

**Figure 6:**
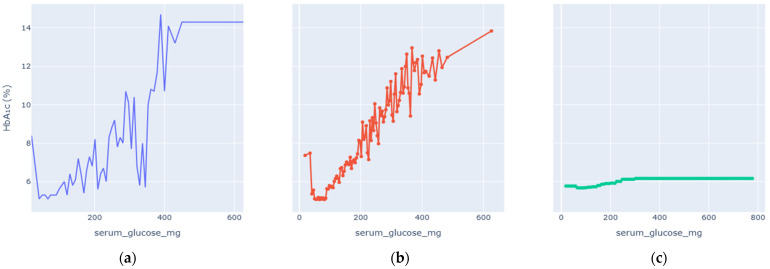
Serum Glucose vs. HbA1c Levels PDP. (**a**) Observed relationship between Serum Glucose and measured HbA1c values. (**b**) Custom partial dependence plot showing model-predicted HbA1c across the effective Serum Glucose range using robust aggregation. (**c**) Default partial dependence plot computed over the full feature range.

A final model validation was performed using the 40-feature LGBMRegressor to predict HbA1c across all folds. The results are summarized through a quantile–quantile plot as presented in Figure 5. Because predictions are aggregated across all cross-validation folds, Figure 6 represents population-level out-of-sample performance rather than a single test-set trajectory.

The predicted values followed the reference diagonal closely, demonstrating strong agreement between observed and predicted HbA1c. The model performed robustly across the entire HbA1c distribution, with minimal deviation at the upper quantiles as explained in Table 6.

These experiments demonstrate that the proposed hybrid feature-selection and stratified-cross-validated framework achieves accurate, efficient, and interpretable prediction of HbA1c using only 40 features.

## 4. Discussion

The final LightGBM regression model, optimized through the integrated ICS + RFECV feature-selection pipeline, was analyzed for interpretability using feature importance ranking, Partial Dependence Plots (PDPs), and SHAP (Shapley Additive Explanation) analysis. This section aims to evaluate the transparency of the model’s internal reasoning and validate that the identified patterns are consistent with the physiological mechanisms underlying HbA1c regulation.

The emphasis on explainability is central to the framework’s third objective—the development of a transferable and explainable modeling architecture that enhances trust, interpretability, and usability in biomedical research and healthcare systems.

### 4.1. Feature-Importance Analysis

It is important to distinguish between feature relevance used during the selection phase and feature importance derived from the trained model. During feature selection, relevance metrics (e.g., correlation-based ranking in ICS and wrapper-based RFECV) were used to identify informative candidates while reducing redundancy. In contrast, post hoc explainability analyses reflect how the final trained model utilizes the selected features to generate predictions. The observed consistency between highly ranked features in the selection phase and dominant contributors in the explainability analysis provides additional validation of the stability and physiological coherence of the selected feature set, while differences highlight non-linear interactions learned by the model.

As presented in Table 7, feature importance was extracted from the final LightGBM model using gain-based metrics averaged across the five cross-validation folds. The analysis revealed a coherent subset of biochemical and physiological indicators that consistently contributed most to the prediction of HbA1c levels. These features represent the key variables explored in the subsequent Partial Dependence analyses.

These predictors represent distinct physiological dimensions—glucose metabolism, adiposity, cardiovascular regulation, and protein status—that jointly influence long-term glycemic control. Their consistent appearance across folds confirms model stability and clinical coherence, reinforcing the framework’s interpretability objective.

### 4.2. Partial-Dependence Analysis

**Figure 7:**
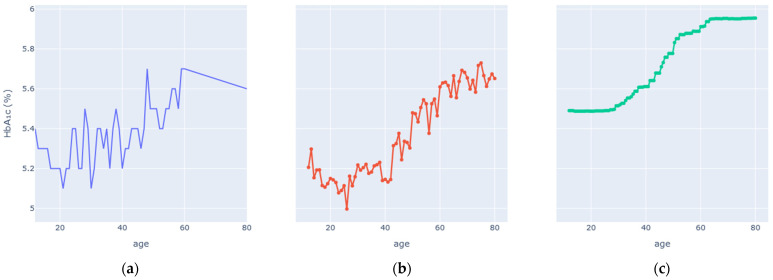
Age vs. HbA1c Levels PDP. (**a**) Observed relationship between Age and measured HbA1c values. (**b**) Custom partial dependence plot showing model-predicted HbA1c across the effective Age range using robust aggregation. (**c**) Default partial dependence plot computed over the full feature range.

**Figure 8:**
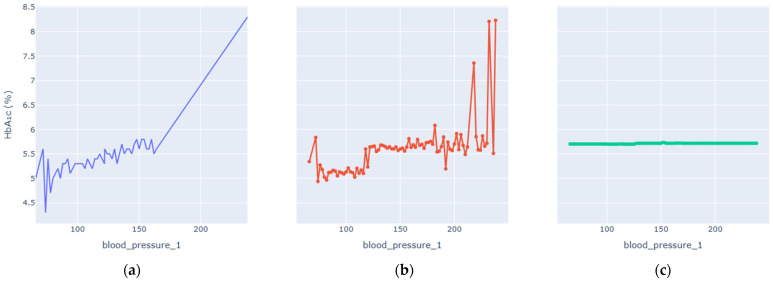
Blood Pressure vs. HbA1c Levels PDP. (**a**) Observed relationship between Blood Pressure and measured HbA1c values. (**b**) Custom partial dependence plot showing model-predicted HbA1c across the effective Blood Pressure range using robust aggregation. (**c**) Default partial dependence plot computed over the full feature range.

**Figure 9:**
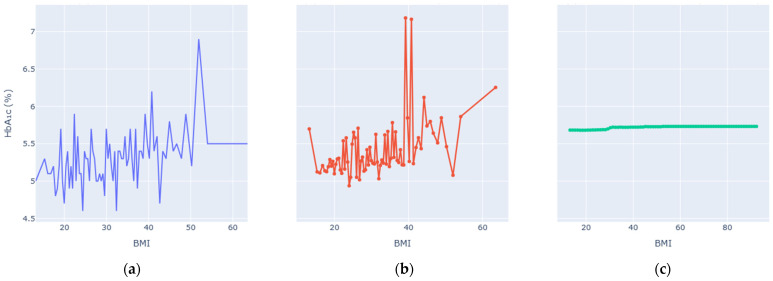
Partial dependence analysis for BMI. (**a**) Observed relationship between BMI and measured HbA1c values. (**b**) Custom partial dependence plot showing model-predicted HbA1c across the effective BMI range using robust aggregation. (**c**) Default partial dependence plot computed over the full feature range.

**Figure 10:**
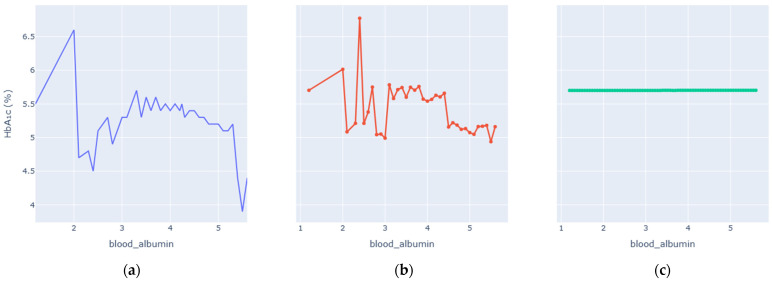
Blood Albumin vs. HbA1c Levels PDP. (**a**) Observed relationship between Blood Albumin and measured HbA1c values. (**b**) Custom partial dependence plot showing model-predicted HbA1c across the effective Blood Albumin range using robust aggregation. (**c**) Default partial dependence plot computed over the full feature range.

**Figure 11:**
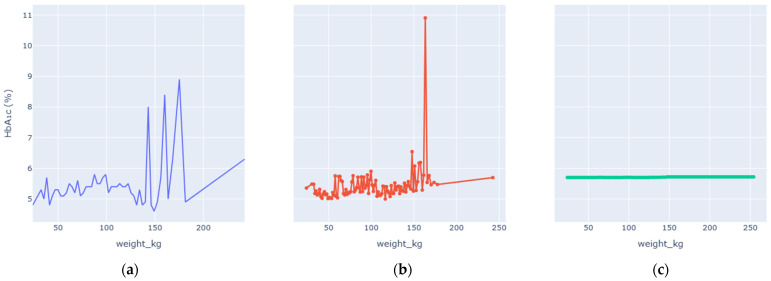
Weight vs. HbA1c Levels PDP. (**a**) Observed relationship between Weight and measured HbA1c values. (**b**) Custom partial dependence plot showing model-predicted HbA1c across the effective Weight range using robust aggregation. (**c**) Default partial dependence plot computed over the full feature range.

Enhanced Partial Dependence Plots were generated to visualize how each key variable influences predicted HbA1c while averaging over all others. The EPDPs isolate meaningful physiological trends by smoothing outliers and clarifying nonlinear behaviors. Figure 6, Figure 7, Figure 8, Figure 9, Figure 10 and Figure 11 present the refined relationships for the major predictors.

Two forms of partial dependence visualization are presented: a default partial dependence plot (Default PDP) and a custom-designed partial dependence plot (Custom PDP). The Default PDP follows the standard implementation, averaging model predictions across the full feature range, which may be influenced by sparse regions, extreme values, and uneven data density. In contrast, the Custom PDP restricts analysis to the effective interquartile range of each feature, applies uniform sampling within data-supported regions, and aggregates predictions using robust statistics. This approach explicitly suppresses outlier-driven effects, improves stability, and facilitates clearer comparison between observed empirical trends and model-learned relationships. As a result, the Custom PDP provides more clinically interpretable insights for population-level health data while preserving the underlying predictive behavior of the model. The comparison highlights how the custom PDP improves interpretability by reducing the influence of sparse or extreme values.

Serum glucose showed a strong, monotonic positive correlation with HbA1c predictions. The enhanced PDP highlighted a smooth upward trajectory: HbA1c values rose slowly at normal glucose concentrations (<5 mmol/L) and increased steeply beyond the pre-diabetic range. A gentle plateau at extreme values suggested physiological saturation of hemoglobin glycation rather than model instability. This confirms that the model accurately reproduces the biochemical linkage between chronic hyperglycaemia and HbA1c accumulation.

Age exhibited a gradual, nonlinear increase in predicted HbA1c. Variability was higher among younger individuals, reflecting lifestyle heterogeneity, whereas after middle age (≈45 years), the slope became steadily positive. A modest plateau beyond 70 years implies stabilization or competing effects of aging physiology. The enhanced PDP clarified this trend by filtering noise, demonstrating that age contributes a consistent but secondary effect relative to biochemical markers.

Blood pressure displayed a threshold-dependent relationship with HbA1c. At normal systolic levels (<120 mm Hg), the curve remained flat, indicating minimal glycemic impact. Above the pre-hypertensive threshold, HbA1c increased gradually, with a pronounced rise beyond 140 mm Hg, reflecting cardiovascular-metabolic coupling typical of metabolic syndrome. The enhanced PDP removed spurious spikes and produced a physiologically realistic progression from normotension to hypertension-associated glycemic stress.

At low BMI (<20 kg/m^2^), HbA1c varied little, but between 25 and 30 kg/m^2^ the curve steepened sharply, reflecting the metabolic transition from overweight to obesity. Above 35 kg/m^2^, the slope flattened, indicating that additional weight confers diminishing incremental glycemic effect—consistent with insulin-resistance saturation. The enhanced PDP reduced extreme outlier noise and highlighted this physiologically credible relationship between adiposity and long-term glucose control.

At typical concentrations (~4 g/dL), predicted HbA1c remained stable. Mild deviations at low or high albumin levels corresponded to subtle changes in predicted HbA1c, possibly reflecting nutritional status, hepatic function, or systemic inflammation rather than direct glycemic mechanisms. The enhanced PDP smoothed abrupt oscillations seen in the default version, suggesting that albumin’s effect is secondary and context-dependent but physiologically interpretable.

Weight alone showed limited direct influence on HbA1c predictions once BMI and waist-related measures were accounted for. The EPDP revealed scattered variability with a faint upward slope at extreme weights. This supports the notion that body composition, not absolute weight, is the more meaningful determinant of metabolic risk. Enhanced smoothing removed random fluctuations and confirmed the absence of spurious correlations.

### 4.3. SHAP Summary and Interaction Analysis

SHAP decomposition quantified each feature’s contribution to individual predictions. Positive SHAP values for serum-glucose and BMI corresponded to upward HbA1c shifts, whereas lower BMI and normal blood pressure exerted negative (protective) effects. The distribution of SHAP values was balanced across folds, indicating fairness and model consistency. Exploratory SHAP-interaction analysis revealed:

- Synergy between serum glucose and BMI, amplifying HbA1c in obese hyperglycaemic individuals.
- Age–BMI modulation, showing that older subjects experienced stronger BMI-related effects.
- Minor interaction between albumin and blood pressure, likely reflecting hydration-status interplay.

These findings confirm that the model captures complex but clinically meaningful dependencies across metabolic domains.

### 4.4. Model Coherence and Statistical Validation

Residual diagnostics showed homoscedasticity and near-normal error distribution (Shapiro–Wilk *p* > 0.05). The QQ-plot in Figure 7 demonstrated close alignment between predicted and observed HbA1c quantiles, confirming proper calibration. The explainability outputs verify that the 40-feature LightGBM model maintains strong predictive performance while remaining physiologically transparent and statistically robust, as presented in Table 8.

The explainability analysis demonstrates that the proposed HbA1c-prediction framework is accurate, interpretable, and physiologically grounded. It successfully identifies well-known glycemic drivers while uncovering secondary metabolic influences, thereby achieving transparency without sacrificing performance. By combining high predictive power (R^2^ ≈ 71.6%) with a reduced, interpretable feature set (40 variables), the model establishes a reproducible, cost-efficient, and clinically coherent foundation for next-generation AI-assisted metabolic assessment.

External validation is an important next step to strengthen the generalizability of the proposed HbA1c regression framework. While this study focuses on NHANES to establish a population-scale, interpretable, and cost-aware modeling pipeline, future work will evaluate the trained models and the full workflow (including preprocessing, imputation, ICS + RFECV feature selection, and stratified regression cross-validation) on independent public cohorts that contain HbA1c and overlapping clinical or demographic predictors. Such cross-dataset validation will help quantify transportability across different measurement protocols and populations. Where feature sets differ, we will use harmonized variable mappings and report performance using a consistent metric suite (e.g., R^2^, MAE, MSE, and MAPE), thereby providing a transparent benchmark of external validity beyond NHANES.

## 5. Conclusions

In this study, we developed and validated a novel machine-learning framework for continuous HbA1c estimation using routinely available clinical, biochemical, and anthropometric variables from a large population-based survey dataset. By progressively refining the modeling pipeline through four experiments, we demonstrated that a gradient-boosting algorithm (LGBMRegressor) combined with a two-stage feature-selection approach (Incremental Correlation Selection followed by RFECV) achieved robust predictive accuracy (R^2^ ≈ 71.6%) while limiting the number of required predictors to just 40 features. These results demonstrate that substantial dimensionality reduction can be achieved without compromising predictive performance, thereby addressing the dual requirements of accuracy and cost-efficiency. Our proposed model is not a black box. Through feature importance ranking, enhanced partial dependence plots, and SHAP interaction analyses, we confirmed that the model’s behavior aligns with physiological mechanisms of glycemic, control-linking fasting glucose, adiposity, vascular indicators, and protein status to long-term hemoglobin glycation. This alignment between model behavior and physiological relevance directly supports the interpretability objective of the proposed framework. Beyond prediction accuracy, the integrated explainability analyses provide clinically meaningful insights and support transparent model interpretation, which is essential for population-level analysis and secondary use in epidemiological research. While the framework can be applied post-training to estimate missing HbA1c values, this functionality is positioned as a practical extension rather than the primary contribution. Based on cross-sectional data and requiring further external and longitudinal validation, the reported findings demonstrate the feasibility of combining multi-stage feature selection, stratified regression validation, and explainability by design into a unified modeling pipeline. This study has several limitations. First, NHANES is a repeated cross-sectional survey rather than a longitudinal cohort; therefore, the model captures associations with HbA1c rather than causal relationships or within-person trajectories. By integrating accuracy, efficiency, and transparency, this work provides an evidence-based blueprint for scalable and interpretable biomarker estimation, with potential applicability to other chronic disease markers in future studies.

## Figures and Tables

**Table 1:** List of abbreviations.

Symbol	Abbreviation	Symbol	Abbreviation
AAP	American Academy of Pediatrics	IoT	Internet of Things
ADA	American Diabetes Association	IQR	Interquartile Range
ANN	Artificial Neural Network	KNN	K-Nearest Neighbor
APR	Area Under the Precision–Recall	LIME	Local Interpretable Model-agnostic Explanations
AUC	Area Under the Curve	NHANES	National Health and Nutrition Examination Survey
Bi-LSTM	Bidirectional Long Short-Term Memory	NLR	Nonlinear Regressor
BRFSS	Behavioral Risk Factor Surveillance System	PIMA	Pima Indians Diabetes dataset
CatBoost	Category Boosting	PoC	Point-of-care
CDC	Centers for Disease Control and Prevention	PSO	Particle Swarm Optimization
CDSS	Clinical Decision Support System	RCT	Randomized Controlled Trial
CNN	Convolutional Neural Network	RF	Random Forest
DDI	Drug–Drug Interaction	RFE	Recursive Feature Elimination
DNN	Deep Neural Network	RFECV	Recursive Feature Elimination with Cross-Validation
EHR	Electronic Health Record	ROC-AUC	Receiver Operating Characteristic—Area Under the Curve
EPDPs	Enhanced Partial Dependence Plots	SHAP	SHapley Additive exPlanations
FAERS	FDA Adverse Event Reporting System	SMOTE	Synthetic Minority Over-sampling Technique
HbA1c	Glycated hemoglobin	SVM	Support Vector Machine
ICS	Incremental Correlation Selection	XGBoost	eXtreme Gradient Boosting

**Table 2:** Base Model Performance Comparison (*n* = 124 features).

Model Name	R^2^ (%)	MAE	MSE	MAPE	Time (ms)
ExtraTreesRegressor	64.63%	0.367182	0.378722	6.064782	29.7207
RandomForestRegressor	67.95%	0.352481	0.343179	5.860661	49.6841
ElasticNetCV	65.23%	0.37634	0.372287	6.272908	13.6519
RidgeCV	65.28%	0.376941	0.371686	6.285937	4.10772
HuberRegressor	63.88%	0.370616	0.386698	6.086151	13.7874
LinearSVR	63.76%	0.367622	0.387988	6.016995	22.7004
LinearRegression	65.18%	0.377224	0.372781	6.290938	0.98317
XGBRegressor	71.32%	0.33429	0.30711	5.55819	6.82974
HistGradientBoostingRegressor	71.26%	0.33521	0.30773	5.57414	6.37609
**LGBMRegressor**	**71.40%**	**0.33456**	**0.30622**	**5.56662**	**5.12374**

**Table 3:** Performance with different imputation strategies (*n* = 124 features).

Input Strategy	R^2^ (%)	MAE	MSE	MAPE
None	71.40%	0.334557	0.306219	5.566623
mean	71.38%	0.334208	0.306471	5.557417
median	71.29%	0.334316	0.307371	5.559426
**Most_frequent**	**71.45%**	**0.333944**	**0.305706**	**5.554814**

**Table 4:** ICS Experiment Results (LGBMRegressor + Most Frequent Imputation).

Model Name	R^2^ (%)	MAE	MSE	MAPE
LGBMRegressor: Most Frequent	71.49%	0.33475	0.305271	5.570256

**Table 5:** Top RFECV ranking results.

R^2^ (%)	MAE	MSE	MAPE	No. Features	Rank
71.61%	0.333959	0.303977	5.556288	40	1
71.54%	0.334598	0.304761	5.567019	92	2
71.53%	0.334508	0.304786	5.563803	39	3

**Table 6:** Summary of experimental outcomes.

Experiment	Key Objective	Optimal Result	Key Outcome
**Exp 1**	Model benchmarking	LGBMRegressor (R^2^ = 71.40%)	Identified best model
**Exp 2**	Imputation strategy comparison	Most Frequent (R^2^ = 71.45%)	Chosen imputation scheme
**Exp 3**	Incremental Correlation Selection	114 features, R^2^ = 71.49%	Defined high-signal subset
**Exp 4**	Combined ICS + RFECV	40 features, R^2^ = 71.61%	Final optimal feature set

**Table 7:** Feature-Importance for LightGBM model.

Feature	Feature Meaning
LBDSGLSI	Fasting Serum Glucose (mmol/L)
serum_glucose_mg	Fasting Serum Glucose (mg/dL)
age	Participant Age (years)
blood_pressure_1	Systolic Blood Pressure (mm Hg)
BMI	Body-Mass Index (kg/m^2^)
Blood_albumin	Blood Albumin (g/dL)
weight	Body Weight (kg)

**Table 8:** Summary of explainability outcomes.

Analytical Aspect	Observation	Interpretation
Feature Importance	Key predictors span glucose metabolism, adiposity, vascular, and protein markers	Captures holistic metabolic state
EPDP Trends (Figure 6, Figure 7, Figure 8, Figure 9, Figure 10 and Figure 11)	Smooth, clinically plausible monotonic or nonlinear patterns	Reflect genuine physiological relations
SHAP Contributions	Balanced additive effects across features and individuals	Confirms model fairness and stability
Feature Interactions	Nonlinear synergy among metabolic and anthropometric variables	Mirrors real-world cardiometabolic coupling
Model Validation	Stable residuals and distributional alignment	Confirms statistical reliability and trustworthiness

## Data Availability

The data used in this study are publicly available from the National Health and Nutrition Examination Survey (NHANES) at https://wwwn.cdc.gov/nchs/nhanes/. Last access 29 December 2025.

## Appendix A

Table A1 summarizes the features exhibiting the largest signed Pearson correlation coefficients with HbA1c (LBXGH). For example, the biochemistry variable *LBDSGLSI* (fasting serum glucose in mmol/L) and *serum_glucose_mg* (fasting glucose in mg/dL) both correlate with HbA1c at Pearson r ≈ 0.7820, Spearman ≈ 0.4887, Kendall ≈ 0.3613 (*p* < 0.001). Other notable features include age (demographic; r ≈ 0.3297, Spearman ≈ 0.5019), waist circumference (body-measure; r ≈ 0.2868, Spearman ≈ 0.3812), and osmolality *LBXSOSSI* (biochemistry; r ≈ 0.2640, Spearman ≈ 0.2506). Blood-pressure stages and BMI also showed moderate correlations (e.g., blood_pressure_1: r ≈ 0.2352; BMI: r ≈ 0.2284). Blood pressure variables include multiple measurements obtained during a single examination visit, consistent with the NHANES protocol. Specifically, up to three systolic and diastolic blood pressure readings are recorded per participant to account for measurement variability. These repeated measurements were retained as separate features rather than averaged, allowing the model to capture intra-visit variability in blood pressure.

**Table A1:** Comprehensive Evaluation of health-related variables and a target health outcome.

Feature	Pearson	Spearman	Kendall	*p*-Value	Feature Type
LBDSGLSI	0.7820	0.4887	0.3613	0	Biochemistry
serum_glucose_mg	0.7820	0.4887	0.3613	0	Biochemistry
age	0.3297	0.5019	0.3495	0	demographic
waist_circum	0.2868	0.3812	0.2650	0	BodyMeasures
LBXSOSSI	0.2640	0.2506	0.1785	0	Biochemistry
blood_pressure_1	0.2352	0.3412	0.2395	0	BloodPressure
blood_pressure_2	0.2324	0.3316	0.2330	0	BloodPressure
BMI	0.2284	0.3178	0.2196	0	BodyMeasures
blood_pressure_3	0.2246	0.3201	0.2249	0	BloodPressure
LBXSTR	0.2131	0.2623	0.1817	0	Biochemistry
LBDSTRSI	0.2131	0.2623	0.1817	0	Biochemistry
LBXSCLSI	−0.2118	−0.1380	−0.0995	0	Biochemistry
people_over_60_in_house	0.1999	0.3275	0.2636	4.0543 × 10^−176^	demographic
blood_albumin	−0.1978	−0.2470	−0.1788	0	Biochemistry
LBDSALSI	−0.1978	−0.2470	−0.1788	0	Biochemistry
weight_kg	0.1939	0.2536	0.1748	0	BodyMeasures
arm_circum	0.1936	0.2590	0.1789	0	BodyMeasures
LBXSBU	0.1869	0.2460	0.1758	0	Biochemistry
LBDSBUSI	0.1869	0.2460	0.1758	0	Biochemistry
BPACSZ	0.1832	0.2434	0.1951	4.8491 × 10^−229^	BloodPressure
cuff_max_inflation	0.1778	0.3469	0.2588	2.0971 × 10^−215^	BloodPressure
LBDSGBSI	0.1684	0.1866	0.1329	6.2791 × 10^−257^	Biochemistry
LBXSGB	0.1684	0.1866	0.1329	6.2791 × 10^−257^	Biochemistry
URDACT	0.1460	0.1854	0.1279	1.995 × 10^−168^	AlbuminCreatinine
leg_length	−0.1449	−0.1624	−0.1120	1.3423 × 10^−183^	BodyMeasures
LBXSNASI	−0.1364	−0.0162	−0.0110	2.173 × 10^−169^	Biochemistry
LBDHDDSI	−0.1336	−0.1414	−0.0980	5.6343 × 10^−163^	CholesterolHDL
LBDHDD	−0.1336	−0.1414	−0.0980	6.6601 × 10^−163^	CholesterolHDL
DMDEDUC2	−0.1299	−0.1543	−0.1165	1.7701 × 10^−146^	demographic
URXUMA	0.1254	0.1321	0.0911	4.9808 × 10^−145^	AlbuminCreatinine
URXUMS	0.1254	0.1321	0.0911	4.9808 × 10^−145^	AlbuminCreatinine

NHANES variable codes are shown as feature labels; full variable descriptions and definitions are available through the official NHANES variable search portal (https://wwwn.cdc.gov/nchs/nhanes/search/default.aspx accessed on 29 December 2025).

Figure A1 and Figure A2 visually reinforce these findings: Figure A1 displays the signed Pearson correlations for all candidate features, highlighting that a small subset of glucose and anthropometric measures dominate. Figure A2 further reveals that the Biochemistry and Anthropometry categories contribute the lion’s share of the predictive signal, whereas other categories (e.g., environmental or derived ratios) contribute less strongly.

Together, these analyses underpin three key insights: (1) HbA1c variability in this cohort is driven by a relatively small number of robust, routinely measured clinical and biochemical indicators; (2) many of these indicators are easily obtainable (e.g., waist circumference, fasting glucose), supporting our goal of cost-efficient modeling; and (3) the distribution of predictive power justifies our selection of a compact, interpretable 40-feature set—enabling high accuracy without over-reliance on obscure or high-cost measures.
